# Fisetin-Mediated Perturbations of Membrane Permeability and Intracellular pH in *Candida albicans*

**DOI:** 10.4014/jmb.2311.11027

**Published:** 2024-01-10

**Authors:** Younhee Kim

**Affiliations:** Department of Korean Medicine, Semyung University, Jecheon 27136, Republic of Korea

**Keywords:** Antifungal, *Candida albicans*, BCECF, intracellular pH, membrane permeability, fisetin

## Abstract

The antifungal activity of fisetin against *Candida albicans* is explored, elucidating a mechanism centered on membrane permeabilization and ensuing disruption of pH homeostasis. The Minimum Inhibitory Concentration (MIC) of fisetin, indicative of its interaction with the fungal membrane, increases in the presence of ergosterol. Hoechst 33342 and propidium-iodide staining reveal substantial propidium-iodide accumulation in fisetin-treated *C. albicans* cells at their MIC, with crystal violet uptake assays confirming fisetin-induced membrane permeabilization. Leakage analysis demonstrates a significant release of DNA and proteins in fisetin-treated cells compared to controls, underscoring the antifungal effect through membrane disruption. Green fluorescence, evident in both the cytoplasm and vacuoles of fisetin-treated cells under BCECF, AM staining, stands in contrast to controls where only acidic vacuoles exhibit staining. Ratiometric pH measurements using BCECF, AM reveal a noteworthy reduction in intracellular pH in fisetin-treated cells, emphasizing its impact on pH homeostasis. DiBAC_4_(3) uptake assays demonstrate membrane hyperpolarization in fisetin-treated cells, suggesting potential disruptions in ion flux and cellular homeostasis. These results provide comprehensive insights into the antifungal mechanisms of fisetin, positioning it as a promising therapeutic agent against *Candida* infections.

## Introduction

*Candida albicans*, a prominent fungal pathogen causing bloodstream infections, represents a significant threat to human health. In recent times, there has been a discernible shift towards non-albicans *Candida* species, including *C. glabrata*, *C. parapsilosis*, and *C. tropicalis* [1]. The incidence of lethal systemic candidiasis has notably increased, primarily due to the growing numbers of critically ill or immunocompromised patients. Despite extensive research into new antifungal medications, the options for treating candidiasis remain limited, partly due to the eukaryotic nature of fungi analogous to human cells and the emergence of drug-resistant yeast strains, a consequence of the widespread and prolonged use of antifungal agents [2]. Hence, there is an urgent necessity to explore novel antifungals that are both safe and effective against *Candida* infections.

At present, the primary targets of available antifungal drugs for treating systemic fungal infections include the fungal cell membrane, cell wall, and nucleic acid synthesis. Amphotericin B, a polyene, interacts with ergosterol, leading to membrane permeabilization. This interaction results in the formation of an ion channel-like complex, causing the leakage of ions and small organic molecules and ultimately leading to the death of yeast cells [3]. Azoles, a class of antifungal drugs targeting the fungal cell membrane, act by inhibiting ergosterol biosynthesis at the 14α-demethylation step. This inhibition leads to ergosterol depletion and the accumulation of sterol precursors, including 14α-methylated sterols. Consequently, this process results in the formation of a plasma membrane with altered structure and function [4]. Another notable drug targeting the cell membrane is folimycin, an inhibitor of plasma membrane H^+^-ATPase. Folimycin disrupts the electrochemical proton gradient, inducing changes in intracellular pH. Bafilomycin operates by inhibiting vacuolar H^+^-ATPase (V-ATPase), effectively blocking the acidification of intracellular organelles [5]. Echinocandins, inhibitors of (1,3)-β-glucan biosynthesis, target a major component of fungal cell walls [6]. Additionally, nucleotide analogs, such as flucytosine, exert their antifungal effects by inhibiting DNA or RNA synthesis [7].

Natural products have a rich history of serving as sources for therapeutic compounds, providing remedies for various ailments and diseases, including fungal infections. Among these natural compounds, fisetin belongs to the flavonoid family of polyphenols, a secondary metabolite found in various plant parts [8, 9]. Numerous studies underscore the diverse beneficial effects of fisetin, including its neurotrophic [10], anti-inflammatory [11], glucose-regulating [12], antiviral [13], anticarcinogenic [14], anti-allergic [15], and antifungal properties [16-18]. Considering its diverse beneficial effects, including antifungal properties, fisetin represents a compelling candidate for exploring its interactions with yeast cells, particularly regarding intracellular pH regulation.

In yeast cells, the regulation of intracellular pH and vacuolar pH is meticulous, playing a central role in overall pH homeostasis. This regulation affects cellular functions, including enzyme activities and transport kinetics of nutrients and metabolites. Furthermore, the pH gradient across cell membranes is intertwined with cellular energetic mechanisms, including ATP generation [19]. Disruptions in intracellular pH can significantly impact various cellular processes, encompassing ionic homeostasis, equilibrium of reactive oxygen species, apoptosis, cell cycle progression, and cellular mobility.

In the course of this investigation, the study aims to uncover the intricate interactions of fisetin with *C. albicans*, with a specific focus on its influence on membrane permeability and the resulting reduction in intracellular pH. By unraveling these molecular intricacies, the research aims to not only enhance our comprehension of the antifungal mechanisms employed by fisetin but also to offer valuable insights into potential targets for innovative antifungal strategies. These revelations hold strong importance in fortifying our defenses against the persistent threat of fungal infections.

## Materials and Methods

### Microorganisms and Culture Conditions

The strains utilized in this study were *Candida albicans* SC5314 and *C. parapsilosis* ATCC 22019, obtained from the American Type Culture Collection (ATCC, USA), and *C. glabrata* ATCC 2001 (KCCM 50044) and *C. krusei* ATCC 6258 (KCCM 11426) from the Korean Culture Center of Microorganisms (KCCM, Republic of Korea). *C. albicans* SC5314 was employed for routine analysis, while the other strains served as controls. For staining experiments, exponential phase *C. albicans* cells (5 × 10^7^ cells/ml) were cultured in YM broth with either 78 μg/ml fisetin or an equivalent amount of DMSO (0.78%) at 35°C with agitation at 200 rpm for 2 h. Heat-killed cells were prepared by incubating *C. albicans* cells at 90°C for 8 min. Afterwards, *C. albicans* cells were harvested by centrifugation at 12,000 ×*g* for 1 min, washed with PBS (pH 7.2), and subjected to the respective stains.

### Reagents

The following reagents were used in the experiments: Fisetin, DMSO (Dimethyl Sulfoxide), MOPS (Morpholinepropanesulfonic acid), amphotericin B, crystal violet, propidium iodide, Hoechst 33342 and ergosterol were purchased from Sigma, USA. RPMI-1640 medium, Phosphate Buffered Saline (PBS, pH 7.2), Qubit dsDNA BR kit, FungaLight yeast vitality kit, BCECF, AM (2',7'-Bis-(2-Carboxyethyl)-5-(and-6)-Carboxyfluorescein, Acetoxymethyl Ester), and an intracellular pH calibration kit, including valinomycin and nigericin, were procured from Invitrogen, USA. Bradford reagent was purchased from Bio-Rad, USA. Fisetin was dissolved in DMSO to a concentration of 10 mg/ml and stored at -20°C in the dark. Crystal violet (0.1 mg/ml) was dissolved in water, filtered, and stored at -20°C.

### Antifungal Activity Testing

The susceptibility of the tested strain to fisetin was determined using the standard broth microdilution CLSI M27-A3 method [20]. In brief, 2-fold serial dilutions of fisetin or amphotericin B (100 μl) were prepared in RPMI-1640 medium in a 96-well round-bottom microplate. Aliquots of 100 μl of the *Candida* suspension were added to each well, resulting in a final cell density of 1 × 10^3^-5 × 10^3^ cells. Growth and sterility controls using DMSO, which was used as a solvent for fisetin preparation, were included.

### Ergosterol Binding Assay

The ergosterol binding assay was conducted [21] following the CLSI M27-A3 protocol with slight modifications. In a 96-well round-bottom microplate, two-fold dilutions of fisetin were prepared, with or without the addition of 400 μg/ml exogenous ergosterol in separate rows. *C. albicans* cell suspensions were then added to all the wells. MIC values were determined at both 24 and 72 h. Amphotericin B, known for its interaction with membrane ergosterol, was included as a positive control.

### Hoechst 33342 and Propidium-Iodide Double Staining

*C. albicans* cells treated with either fisetin or DMSO or heat-killed cells were stained with 10 μg/ml Hoechst 33342 and 10 μg/ml propidium iodide in 200 μl of PBS for 30 min at 30°C in the dark. After staining, the cell suspension was precipitated by centrifugation at 12,000 ×*g* for 1 min, washed with PBS (pH 7.2), resuspended in PBS (pH 7.2), and observed under a fluorescence microscope equipped with triple RGB filters or a bright-field microscope.

### Crystal Violet-Uptake Assay

The impact of fisetin on membrane permeability was assessed through a crystal violet uptake assay, employing a method based on that described by Vaara and Vaara [22, 23] with minor modifications. *C. albicans* cells in exponential phase were collected by centrifugation at 12,000 ×*g* for 5 min, washed, and suspended in PBS (pH 7.2) to a density of 5 × 10^7^ cells/ml. The cell suspensions were then treated with 1×, 2×, and 4× MIC fisetin at 35°C with shaking at 200 rpm for 30 min. DMSO controls were included for each fisetin treatment.

After the incubation, 800 μl of the cell suspension was centrifuged at 12,000 ×*g* for 5 min, washed with PBS (pH 7.2), and resuspended in 1 ml of PBS (pH 7.2) containing 10 μg/ml crystal violet. The cell suspension was incubated at 30°C with shaking at 200 rpm for 15 min.

Following the incubation, the cells were precipitated by centrifugation at 12,000 ×*g* and 4°C for 20 min, and the supernatant (200 μl) was transferred to a 96-well flat-bottom microplate in quadruplicate. The amount of crystal violet remaining in the supernatant was measured as the absorbance at 590 nm (A_590_) using a spectrofluorometer (Tecan, Austria). The absorbance of the initial crystal violet solution used in the assay was considered as 100%. The percentage of crystal violet uptake was calculated using the following formula: uptake of crystal violet (%) = 100 − (A_590_ of the sample/A_590_ of crystal violet solution) × 100.

### Leakage Assay of Intracellular Materials

Fisetin-induced nucleotide and protein leakage were assessed using fluorometric and spectrophotometric methods, respectively, following the procedure described by Lee and Kim [23]. *C. albicans* cells in the logarithmic phase were harvested, washed, and resuspended in PBS (pH 7.2) to a cell density of 5 × 10^7^ cells/ml. The cell suspensions were then incubated with 78 μg/ml and 156 μg/ml fisetin, respectively, along with DMSO controls for each fisetin treatment, at 35°C with agitation at 200 rpm for 30 or 60 min. After incubation, the cell suspensions (800 μl) were centrifuged at 12,000 ×*g* at 4°C for 20 min, and the supernatants were collected for the analysis of nucleotide and protein leakage.

Nucleotide leakage was evaluated using Qubit dsDNA BR assay kits and a Qubit 4 Fluorometer, following the manufacturer's manual. The fluorescence of the supernatant (20 μl) mixed with working solution (180 μl) was measured in triplicate, and the concentrations of nucleotides in the samples were calculated using the dilution calculator of the Qubit 4 fluorometer. Protein leakage was assessed using Bio-Rad's Bradford assay [24], following the manufacturer's instructions. The supernatant (50 μl) was mixed with diluted Bradford reagent (150 μl) in PBS (pH 7.2) and placed in a 96-well clear flat-bottom microplate in quadruplicate. The absorbance of the samples at 590 nm was measured using a spectrophotometer. The amount of protein leakage was calculated as the A_590_ of the sample minus the A_590_ of the diluted Bradford reagent with PBS (pH 7.2).

### pHrodo^TM^ Red Staining

*C. albicans* cells treated with either fisetin or DMSO or heat-killed cells were stained with 5 μM pHrodo^TM^ Red in 200 μl of PBS (pH 7.2) for 30 min at 30°C in the dark. Following staining, the cell suspension was centrifuged at 12,000 ×*g* for 1 min, washed once more with PBS (pH 7.2), and resuspended in PBS (pH 7.2). Finally, the stained cells were observed under a fluorescence microscope equipped with triple RGB filters or a bright-field microscope.

### BCECF Staining

*C. albicans* cells treated with either fisetin or DMSO or heat-killed cells were stained with 10 μM BCECF, AM in PBS (pH 7.2) for 30 min at 30°C in the dark. After staining, the cell suspension was washed and resuspended in PBS (pH 7.2) and examined using a fluorescence microscope equipped with triple RGB filters or a bright-field microscope.

### Ratiometric Measurement of Intracellular pH

Intracellular pH fluctuations in *C. albicans* cells were assessed using ratiometric pH measurements employing the pH-sensitive fluorescence dye BCECF, AM. The pH-dependent ratio (I_485_/I_460_) was calculated by measuring the emission intensity detected at 535 nm when the dye was excited at 485 nm, relative to the emission intensity at its isosbestic point of 460 nm when excited.

First, BCECF, AM-labeled *C. albicans* cells were prepared as follows: *C. albicans* cells in the exponential phase (1 × 10^8^ cells) were washed with PBS (pH 7.2) and incubated with 10 μM BCECF, AM in PBS (pH 7.2) at a cell density of 5 × 10^7^ cells/ml for 30 min at 30°C in the dark with mild agitation. Subsequently, the cells were harvested, washed, and resuspended in 2.2 ml PBS (pH 7.2), referred to as BCECF, AM-labeled cells in PBS (pH 7.2).

Secondly, a pH standard curve was established using BCECF, AM-labeled cells in PBS (pH 7.2) in conjunction with an intracellular pH calibration kit containing buffers A (pH 4.5), B (pH 5.5), C (pH 6.5), and D (pH 7.5), along with PBS (pH 7.2). Each BCECF, AM-labeled cell suspension (100 μl) was harvested, washed with 500 μl of pH calibration buffer, and then resuspended in 500 μl of pH calibration buffer containing 10 μM valinomycin and 10 μM nigericin. This process was performed in duplicate. Next, 100 μl of the cell suspension was loaded into the wells of a black flat-bottom 96-well plate in quadruplicate and incubated at room temperature in the dark with mild agitation. Fluorescence intensity measurements were taken every 30 min at an excitation wavelength of 485 nm with a bandwidth of 20 nm and an emission wavelength of 535 nm with a bandwidth of 25 nm (I_485_), as well as at 460 nm excitation with a bandwidth of 10 nm and 535 nm emission wavelengths with a bandwidth of 25 nm (I_460_). The ratio of I_485_/I_460_ was plotted against pH, and the data presented depict the mean of the quadruplicate measurements ± standard deviation taken at 4 h.

Third, pH measurement of *C. albicans* cells treated with DMSO or fisetin was carried out with BCECF, AM-labeled *C. albicans* cells in PBS (pH 7.2). Each BCECF, AM-labeled cell suspension (100 μl) was treated DMSO or fisetin at its 1x, 2x, and 4x the MIC at 35°C with agitation at 200 rpm for 30 min. The cells were then harvested, washed with PBS (pH 7.2) twice, and resuspended in 900 μl of PBS (pH 7.2). Following these steps, each cell suspension (200 μl) was transferred into a 96-well black flat-bottom plate in quadruplicate. Fluorescence intensity measurements were taken using a spectrofluorometer 8 min after the cessation of the treatment. The measurements were taken at 485 nm excitation with a bandwidth of 20 nm and 535 nm emission wavelengths with a bandwidth of 25 nm, as well as at 460 nm excitation with a bandwidth of 10 nm and 535 nm emission wavelengths with a bandwidth of 25 nm. Ratiometric pH measurements with BCECF (I_485_/I_460_) were determined by calculating the ratio of emission intensity detected at 535 nm when excited at 485 nm to the emission intensity at its isosbestic point of 460 nm. The provided data illustrate the mean of the quadruplicate measurements ± standard deviation.

### Measurements of Membrane Potential

The effect of fisetin on the membrane potential of *C. albicans* was assessed using DiBAC_4_(3), following the method outlined by Lee and Kim with slight modifications [25]. *C. albicans* cells in the exponential growth phase (1 × 10^7^ cells) were exposed to fisetin at concentrations equal to 1× MIC, 2× MIC, and 4× MIC while maintained at 35°C with continuous shaking at 200 rpm. For each fisetin treatment, control samples with the solvent (DMSO) were included. After a 30-min incubation, the cells were collected, washed with 1 ml of PBS (pH 7.2), and then resuspended in PBS (pH 7.2) containing 20 μg/ml of DiBAC_4_(3). Each cell suspension (200 μl) was placed into individual wells of a 96-well black flat-bottom microplate in quadruplicate, and fluorescence intensity was measured after 6 min using a spectrofluorometer set to excitation at 485 nm with a bandwidth of 20 nm and emission at 520 nm with a bandwidth of 10 nm. The presented data represent the mean of four measurements obtained from two separate, independent experiments.

### Statistical Analysis

Each experiment was repeated at least twice, with triplicate or quadruplicate samples. The results were presented as the mean value accompanied by the standard deviation. SigmaPlot 13.0 was utilized to conduct a statistical analysis comparing the effect of fisetin with the control groups. A significance level of *p* < 0.05 was used to determine statistical significance. Regression analysis was carried out to create the pH standard curve using SigmaPlot 13.0.

## Results

### Fisetin Exhibits Antifungal Activity against Diverse *Candida* Species

The MIC of fisetin used for routine assays of *C. albicans* SC5314 was found to be higher (78 μg/ml) compared to the MIC of amphotericin B (1 μg/ml), indicating that fisetin exhibits lower antifungal activity than amphotericin B, which serves as the positive control (Table 1). The MIC values of fisetin against *C. glabrata* ATCC 2001, *C. krusei* ATCC 6258, and *C. parapsilosis* ATCC 22019 were 39 μg/ml, 39 μg/ml, and 19.5 μg/ml, respectively. The MIC values of amphotericin B against the tested *Candida* species ranged from 0.5 μg/ml to 1 μg/ml.

### Fisetin Binds to *Candida*’ Ergosterol

Ergosterol, the primary sterol in fungal cell membranes, plays a crucial role in regulating membrane permeability, fluidity, and membrane protein integrity [26, 27]. To investigate whether the antifungal activity of fisetin is attributed to its binding with ergosterol, antifungal susceptibility tests were conducted with and without exogenous ergosterol. The purpose of adding exogenous ergosterol was to evaluate if it hinders the binding of fisetin to ergosterol in fungal membranes. The positive control, amphotericin B, was included to illustrate its interaction with ergosterol, disrupting the integrity and permeability of the fungal cell membrane.

In the absence of exogenous ergosterol, the MIC of fisetin increased twofold from 78 μg/ml to 156 μg/ml after 72 h. Conversely, in the presence of exogenous ergosterol, no significant alteration in the MIC of fisetin was observed after 24 h. However, after 72 h, the MIC value of fisetin increased fourfold from 78 μg/ml to 312 μg/ml (Table 2). These findings suggest that an excess of ergosterol competes with fisetin for binding sites, thereby reducing its antifungal effectiveness against *C. albicans* cells. Similarly, in the presence of ergosterol, no change in the MIC of amphotericin B was observed after 24 h. However, the MIC of amphotericin B increased fourfold in the presence of exogenous ergosterol after 72 h. These data suggest that fisetin affects *C. albicans* cell membrane fluidity and permeability through its interaction with ergosterol. Notably, the twofold increase in MIC for fisetin after 72 h in RPMI-1640 medium raises the possibility that fisetin may exhibit fungistatic activity, in contrast to the fungicidal activity seen with amphotericin B.

### Fisetin Changes Membrane Permeability in *Candida*

Given fisetin's proven action on the cell membrane of *C. albicans*, the effect of fisetin was assessed on membrane permeability using propidium iodide accumulation through Hoechst 33342-propidium iodide staining, crystal violet uptake, and leakage of intracellular materials.

Hoechst 33342, a blue fluorescent stain, selectively binds to the minor groove of DNA with AT selectivity, making it effective for use in both live and dead cells. Its small size and lipophilic properties grant it the ability to permeate both live and dead cells. In contrast, propidium-iodide, an intercalating fluorescent dye, emits a red fluorescence signal upon binding to DNA. While live cells with intact membranes typically exclude propidium iodide, it readily permeates dead or dying cells with compromised membranes.

**Fig. 1:**
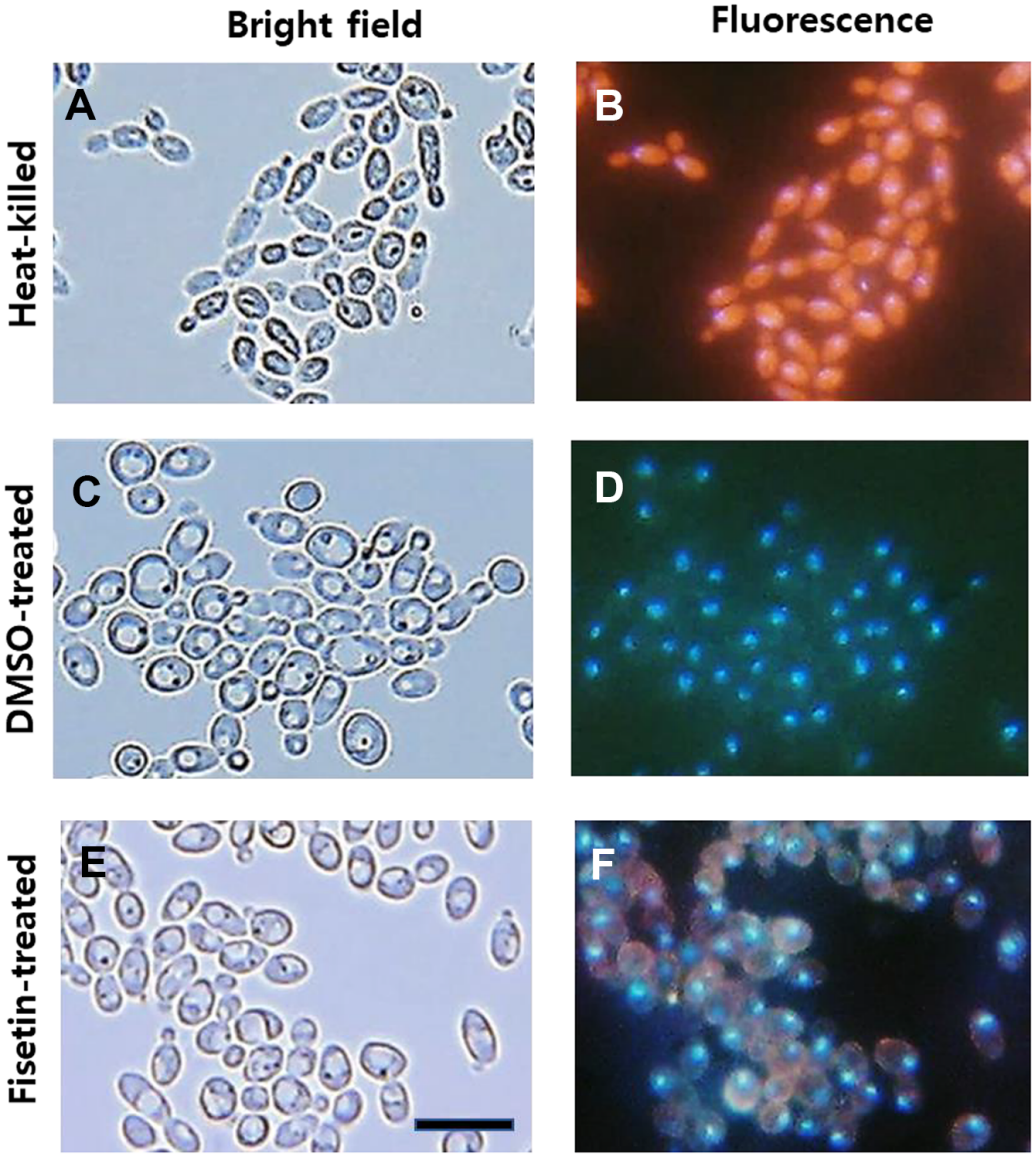
Double staining of *C. albicans* SC5314 cells with Hoechst 33342 and propidium iodide. Heat-killed (**A** and **B**), DMSO-treated (**C** and **D**), and fisetin-treated (**E** and **F**) *C. albicans* cells were double-stained with 10 μg/ml Hoechst 33342 and 10 μg/ml propidium iodide. The cells were observed under a bright-field microscope (**A**, **C**, and **E**) and a fluorescence microscope (**B**, **D**, and **F**). The nuclear region of *C. albicans* cells was stained blue with Hoechst 33342, while cells with damaged membranes were stained red (**B**) and appeared reddish (**F**) with propidium iodide, respectively. Scale bar: 10 μm.

Heat-killed cells (Fig. 1B) exhibited pronounced red staining throughout the cytoplasm, indicative of compromised membranes and, presumably, loss of viability. Moreover, the strong red fluorescence caused by propidium iodide rendered Hoechst 33342 staining in the nucleus less distinct but discernable in this double staining of *C. albicans* SC5314 cells. In contrast, both DMSO-treated (Fig. 1D) and fisetin-treated *C. albicans* cells (Fig. 1F) exhibited blue Hoechst 33342 staining within the nucleus. DMSO-treated control cells, representing viable cells with intact membranes, showed no propidium iodide staining (Fig. 1D). Notably, after a 2-h exposure, fisetin-treated *C. albicans* cells at their MIC showed a substantial accumulation of propidium iodide in the cytoplasm (Fig. 1F), suggesting a potential compromise in membrane integrity and a possible impact on cell viability.

Crystal violet, a lipophilic cation at neutral pH, readily penetrates cells with impaired cell membranes but does not pass through intact cell membranes. Consequently, the crystal violet-uptake assay is a suitable method for studying membrane damage [23]. If fisetin is involved in impairing the cell membrane of *C. albicans*, it would affect the permeability of the cells. Therefore, a crystal violet-uptake assay was conducted to determine whether fisetin influences the membrane permeability of *C. albicans* cells.

**Fig. 2:**
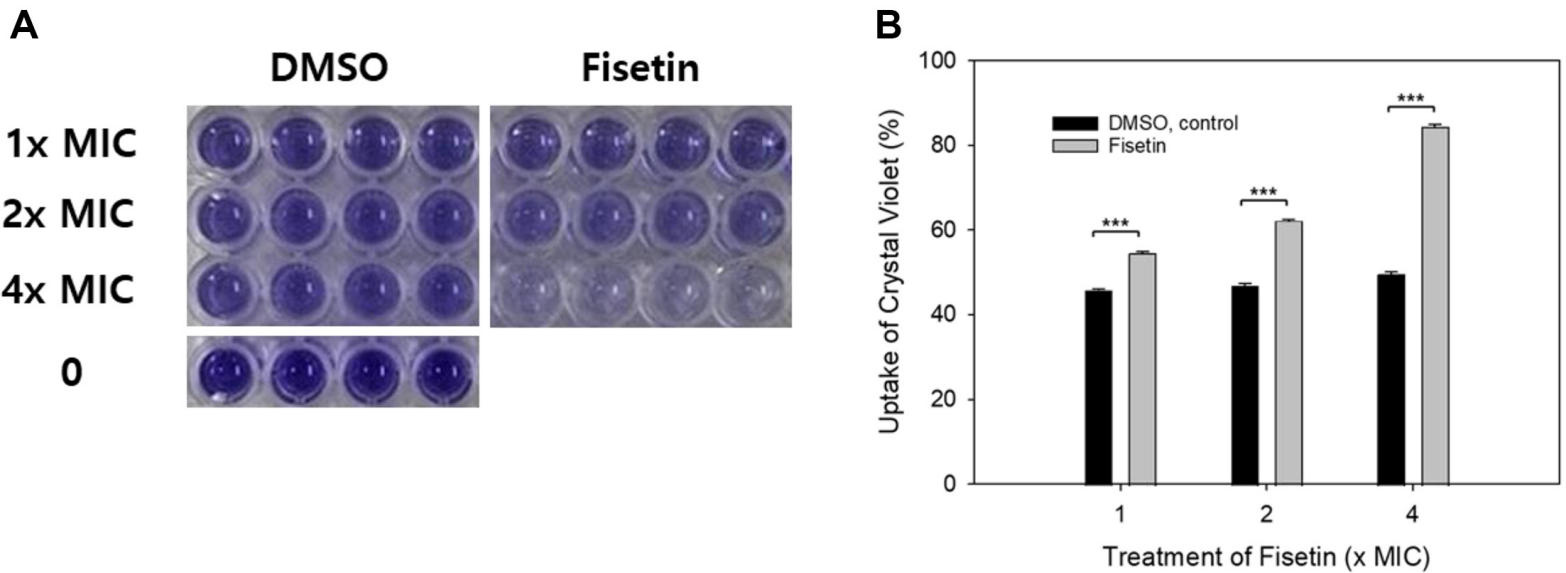
Crystal violet-uptake assay. *C. albicans* SC5314 cells treated with DMSO or fisetin for 30 min were incubated with 10 μg/ml crystal violet in PBS (pH 7.2) for 15 min. The cells were then centrifuged, and the supernatant was transferred to a 96- well flat-bottom plate in quadruplicate as shown (**A**) The amount of crystal violet in the supernatant was measured as the absorbance at 590 nm. The relative uptake of crystal violet in DMSO- or fisetin-treated *C. albicans* cells is presented as the mean ± standard deviation (**B**) Data shown are representative of one of three independent experiments. ***p < 0.001.

After treating *C. albicans* cells with fisetin or an equivalent amount of DMSO for 30 min, the cells were exposed to a crystal violet solution. The cells were then precipitated, and the supernatants were transferred to a 96-well microplate, as shown in Fig. 2A. A noticeable difference in the color of the supernatants was observed between the DMSO control and the fisetin-treated samples, and this difference was concentration-dependent. The percentage of crystal violet uptake by *C. albicans* cells incubated with 1×, 2×, and 4× the MIC of fisetin for 30 min was 54.7%, 62.2%, and 84.3%, respectively. In comparison, the percentage of crystal violet uptake by *C. albicans* cells incubated with the corresponding amounts of DMSO was 45.8%, 46.9%, and 49.6%, respectively (Fig. 2B). The difference between each fisetin-treated sample and the corresponding DMSO control was found to be statistically significant (*p* < 0.001), indicating that fisetin significantly increases the membrane permeability of *C. albicans* cells.

Since fisetin has been shown to alter the membrane permeability of *C. albicans* cells, leakage analysis experiments were carried out to investigate whether treatment with fisetin induces the leakage of nucleotides and proteins.

**Fig. 3:**
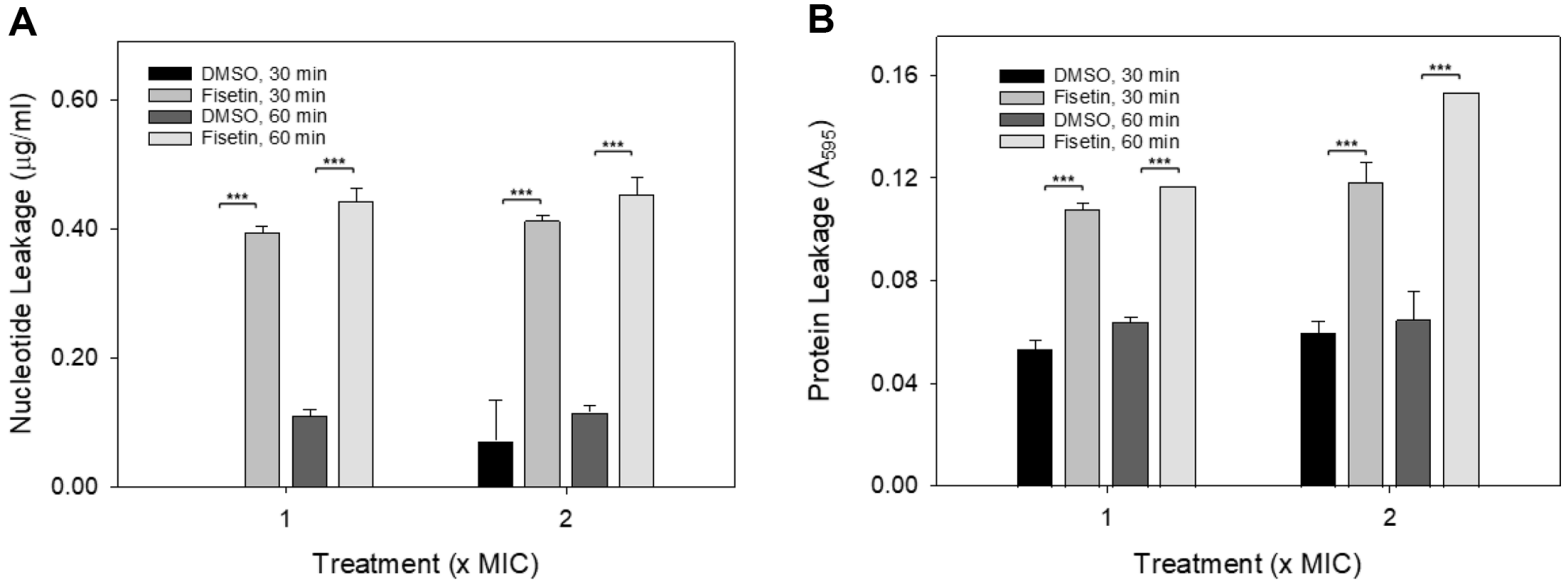
Nucleotide and protein leakage. Exponential-phase *C. albicans* cells were treated with 1× or 2× the MIC of DMSO or fisetin and incubated for 30 or 60 min, respectively. After incubation, the cells were centrifuged and the supernatants were collected for nucleotide leakage analysis (**A**) and protein leakage analysis (**B**) The experiments were performed in triplicate, and representative data from one of the three independent experiments are shown as the mean ± standard deviation. Statistical analysis revealed significant differences between the DMSO control and the fisetin-treated samples. ***p < 0.001.

Nucleotide leakage was analyzed using the Qubit dsDNA BR assay kits, which selectively measure the levels of double-stranded DNA over RNA, as recommended by the manufacturer. After treating *C. albicans* cells with 1× and 2× the MIC of DMSO or fisetin for 30 or 60 min, respectively, the levels of DNA leakage were measured. The results showed DNA leakage of 0.395 μg/ml and 0.412 μg/ml in the cells treated with 1× and 2× MIC of fisetin for 30 min, respectively. In contrast, an undetectable amount of DNA leakage and 0.072 μg/ml of DNA leakage were observed in the *C. albicans* cells treated with equivalent amounts of DMSO (0.78% and 1.56% DMSO, respectively)(Fig. 3A). Statistical analysis revealed a significant difference between the levels of DNA leakage in the fisetin-treated cells and the corresponding DMSO-treated controls (*p* < 0.001). These findings strongly indicate that fisetin treatment leads to the leakage of nucleotides from *C. albicans* cells.

Protein leakage was assessed by measuring the absorbance at 590 nm (A_590_) after mixing the supernatant with the Bradford reagent (Fig. 3B). The A_590_ values for the cells treated with 1× and 2× the MIC of fisetin for 30 min were 0.108 and 0.118, respectively, while the A_590_ values for the cells treated with the equivalent amounts of DMSO were 0.053 and 0.060, respectively. Statistical analysis revealed a significant difference between the A_590_ values of the fisetin-treated cell samples and the corresponding DMSO-treated control cell samples (*p* < 0.001). These findings demonstrate that fisetin treatment leads to noticeable protein leakage from *C. albicans* cells. Based on the results from the crystal violet-uptake assay (Fig. 2) and the analysis of nucleotide and protein leakage (Fig. 3), it can be concluded that fisetin exerts antifungal effects by inducing membrane permeabilization in *C. albicans* cells.

### Fisetin Alters the pH of *Candida* Cells

Maintaining pH homeostasis is vital for yeast cell survival. This study focuses on understanding the impact of fisetin on the membrane permeability of *C. albicans* cells and its potential effect on intracellular pH. Yeast cells possess acidic vacuoles, functionally analogous to lysosomes in animal cells [28]. Reported pH levels within budding yeast indicate a range of 5.6 to 6.1 in vacuoles, contrasting with a cytoplasmic pH of approximately 7.0 [29]. In this section, key findings from the experiment are presented, emphasizing the influence of fisetin on *C. albicans* cells using pHrodo^TM^ Red staining. To investigate fisetin's effect on membrane permeability and potential impact on intracellular pH, pHrodo^TM^ Red, a pH-sensitive dye, was employed.

The staining process, employing a pH-sensitive dye with a pKa value of 6.8, is crucial for revealing relative pH levels in cellular compartments. The dye can show relative pH levels in various cellular compartments, with a pKa value of approximately 6.8, displaying weak fluorescence at higher pH levels that intensifies as pH decreases, as stated by the manufacturer.

**Fig. 4:**
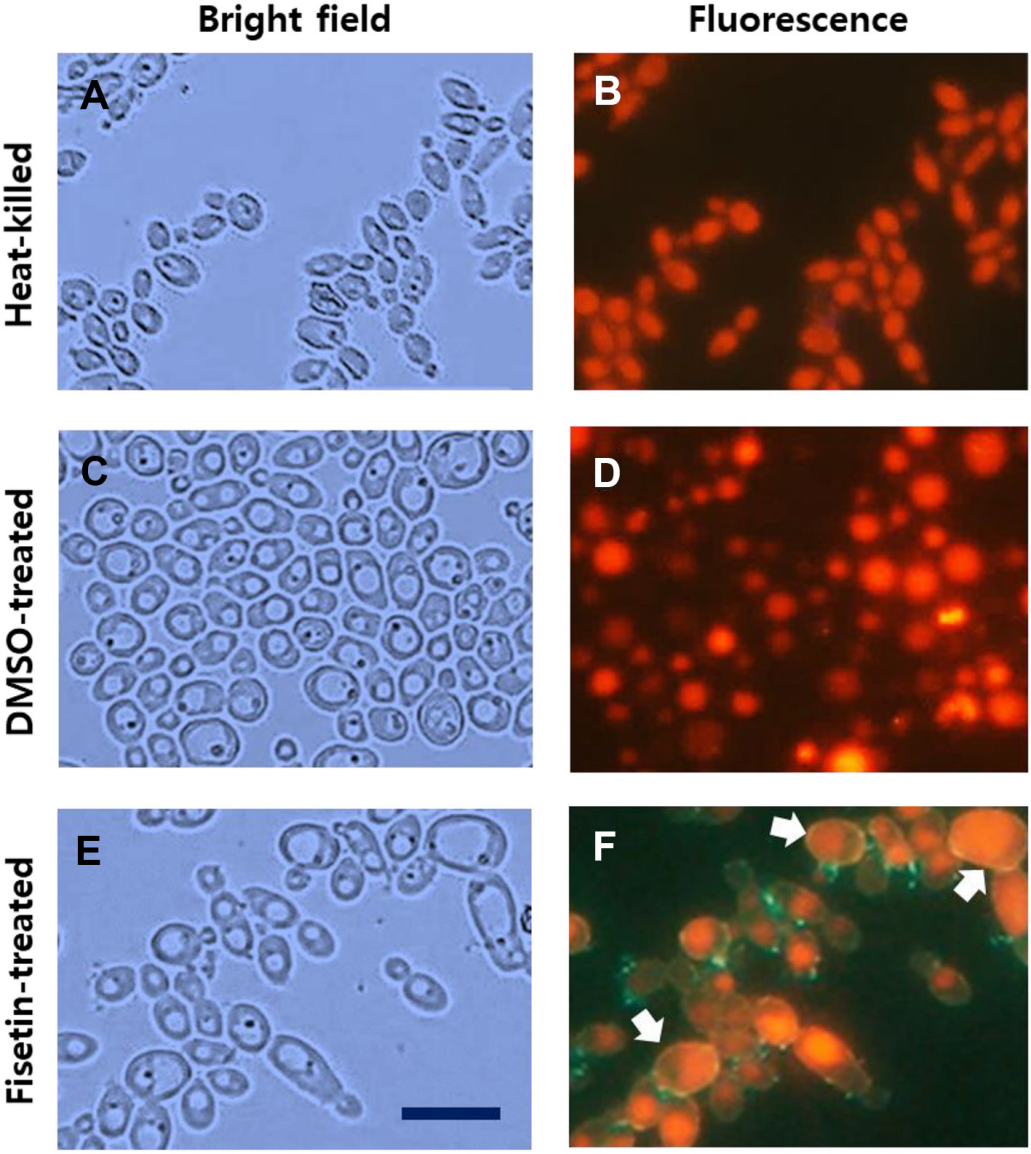
Intracellular pH imaging in *C. albicans* cells. Heat-killed (**A** and **B**), DMSO-treated (**C** and **D**), and fisetintreated (**E** and **F**) *C. albicans* cells were stained with 5 μM pHrodo^TM^ Red, AM intracellular pH indicator using PowerLoadTM Concentrate for 30 min at room temperature and observed using a bright-field microscope (**A**, **C**, and **E**) and a fluorescence microscope (**B**, **D**, and **F**). The distribution and intensity of the dye provide an indication of relative pH in different cellular compartments. Acidic organelles and compartments are fluorescent more brightly. Scale bar: 10 μm.

In the experiment depicted in Fig. 4, heat-killed, DMSO-treated, and fisetin-treated *C. albicans* cells underwent pHrodo^TM^ Red staining and were examined using bright-field and fluorescence microscopy. Most heat-killed *C. albicans* cells exhibited an elongated ovoid shape with a shrunken size. Although vacuoles were detectable in the bright-field image (Fig. 4A), heat-killed cells emitted strong red fluorescence in the vacuole and the whole intracellular region, respectively, signifying a disruption of pH homeostasis. Therefore, the region of red fluorescence appeared elongated ovoid shape (Fig. 4B). In contrast, live cells displayed intense red fluorescence in their acidic vacuoles and weak fluorescence in their neutral cytoplasm (Fig. 4D). The fluorescent region looked round due to the shape of the vacuole (Fig. 4D). Fisetin-treated *C. albicans* cells appeared oval, with enlarged vacuoles, displaying orange fluorescence in vacuoles and dim orange fluorescence in the cytoplasm (Fig. 4F). The orange fluorescence was initially thought to be due to the pH difference, but it was found that the color was due to the autofluorescence of fisetin. It's worth noting that fisetin-treated cells exhibited a greenish appearance with distinct green cell boundaries, attributed to the green autofluorescence of fisetin, which is excited at 345 nm and emits light at 520 nm [30]. This autofluorescence of fisetin adhered to the surface of *C. albicans* cells may complicate data interpretation, making it challenging to distinguish between DMSO- and fisetin-treated cells in terms of fluorescence color and intensity. Nonetheless, these observations suggest that fisetin may influence intracellular pH, especially of the cytoplasm and the vacuole in *C. albicans* cells. Arrows indicate the cells showing strong fluorescence in the vacuole and weak fluorescence in the cytoplasm (Fig. 4F). Additionally, it's important to note that fisetin's antifungal activity might be related to its adherence to the cell components (Fig. 4F) and the formation of aggregates, as often observed in microscopic analyses (Fig. S1).

The influence of fisetin on intracellular pH in *C. albicans* cells is apparent, but the exact direction of this influence, whether it results in an increase or decrease in intracellular pH, requires further investigation. To unravel the pH dynamics within *C. albicans*, BCECF, a well-established pH-sensitive fluorescent dye derived from fluorescein, was employed. BCECF has a pKa of approximately 6.98 and, when modified with AM ester groups, can efficiently penetrate cell membranes. Once inside the cell, nonspecific esterases cleave the lipophilic blocking groups, resulting in a charged form that exhibits reduced leakage from cells, particularly within yeast vacuoles [31].

**Fig. 5:**
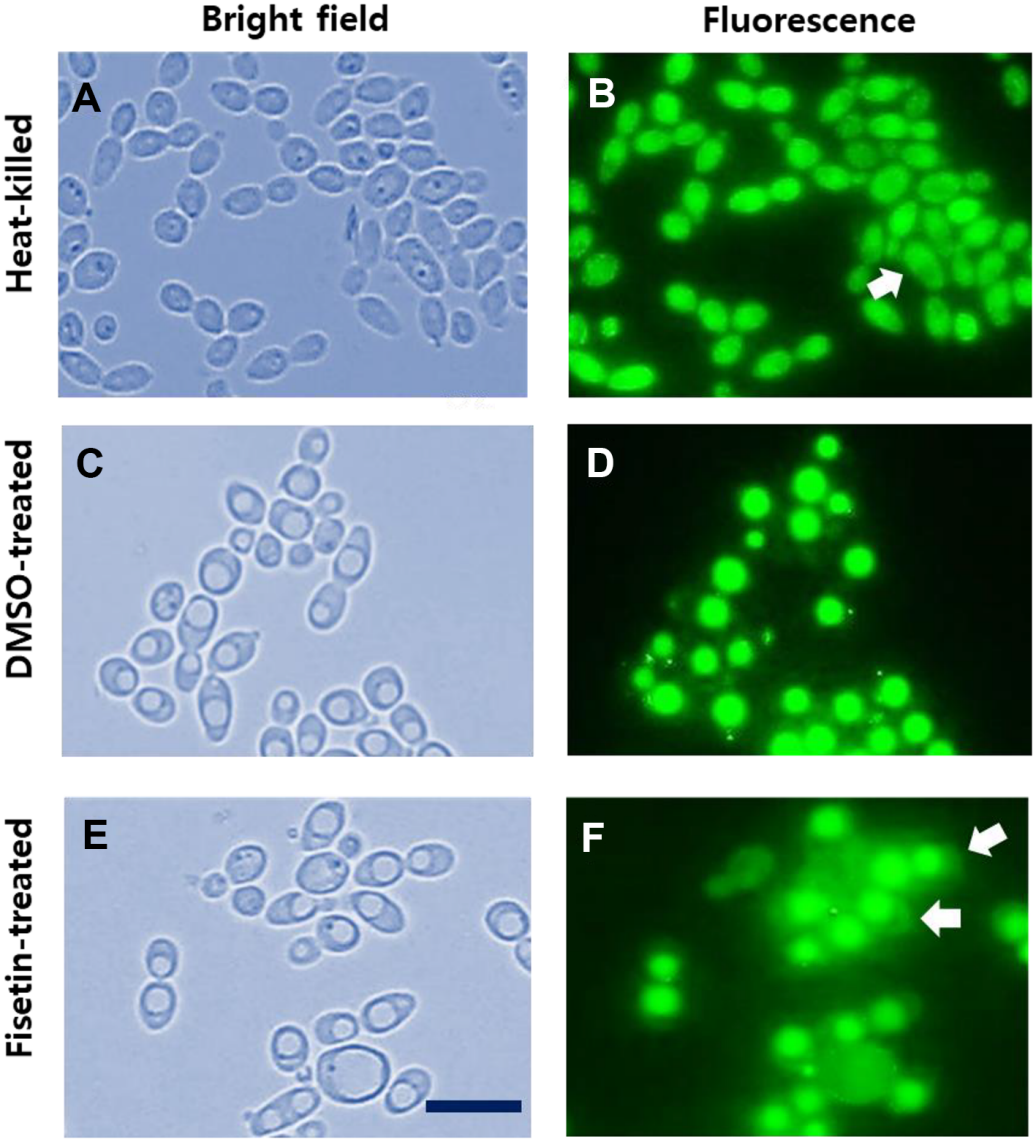
Observation of pH changes with BCECF staining of *C. albicans* cells. Heat-killed (**A** and **B**), DMSO-treated (**C** and **D**), and fisetin-treated (**E** and **F**) *C. albicans* cells were stained with 10 μM BCECF, AM, and examined using a brightfield microscope (**A**, **C**, and **E**) and a fluorescence microscope (**B**, **D**, and **F**), respectively. Scale bar: 10 μm.

As shown in Fig. 5, heat-killed, DMSO-treated, and fisetin-treated *C. albicans* cells underwent BCECF staining. In the bright-field image, heat-killed *C. albicans* cells displayed an elongated ovoid morphology with a shrunken size (Fig. 5A). They emitted relatively uniform green fluorescence throughout the entire intracellular region, but some cells showed strong green and green fluorescence in the vacuole and the cytoplasm, respectively, indicating disrupted pH homeostasis (Fig. 5B). An arrow indicates the cell showing weak fluorescence in the cytoplasm and strong fluorescence in the vacuole due to disrupted pH homeostasis.

In contrast, live *C. albicans* cells exhibited a round ovoid shape with conspicuous vacuoles (Fig. 5C) in a bright-field image, and displayed robust green fluorescence in their acidic vacuoles with a round shape, in contrast to the minimal green fluorescence in the neutral cytoplasm (Fig. 5D).

Significantly, fisetin treatment (Fig. 5E and 5F) resulted in round ovoid cells with enlarged vacuoles, displaying prominent green fluorescence in vacuoles and a modest amount of green fluorescence in the cytoplasm. Intriguingly, some of the cells were almost uniformly stained with BCECF throughout the entire cell area, and vacuolar boundaries were indistinct (Fig. 5F), despite the visibility of vacuoles in the bright-field image (Fig. 5E). Arrows (Fig. 5F) indicate the cells showing the green fluorescence in the cytoplasm, which characteristics are not detectable in live DMSO-treated cells. This suggests that the pH difference between the cytoplasm and vacuole is not discernible, possibly due to damage to the cell membranes, possibly with the vacuolar membranes.

In fisetin-treated cells, the green fluorescence observed in the cytoplasm originates from BCECF free acids, which are formed by the hydrolysis of BCECF, AM by intracellular esterases, as previously described. Since intracellular esterases are primarily localized within the vacuoles in yeast cells, the hydrolysis process takes place inside the vacuoles, generating the charged and fluorescent BCECF free acid. The increase in green fluorescence in the cytoplasm of fisetin-treated cells suggests that BCECF free acids or intracellular esterases are leaking out of the vacuoles and entering the cytoplasm, possibly due to increased permeability of the vacuolar membranes. Alternatively, a decrease in cytoplasmic pH resulting from a damaged cell membrane could activate nonspecific intracellular esterases in the cytoplasm, leading to the formation of BCECF free acids from BCECF, AM. This series of observations elucidates the potential impact of fisetin on intracellular pH dynamics and the integrity of both vacuolar and cell membranes.

BCECF possesses the valuable property of dual-excitation ratiometry, with emission intensity measured at 535 nm when excited at 490 nm, along with an isosbestic point at 440 nm. This property endows BCECF with the capability to precisely assess pH alterations within yeast cells [32].

**Fig. 6:**
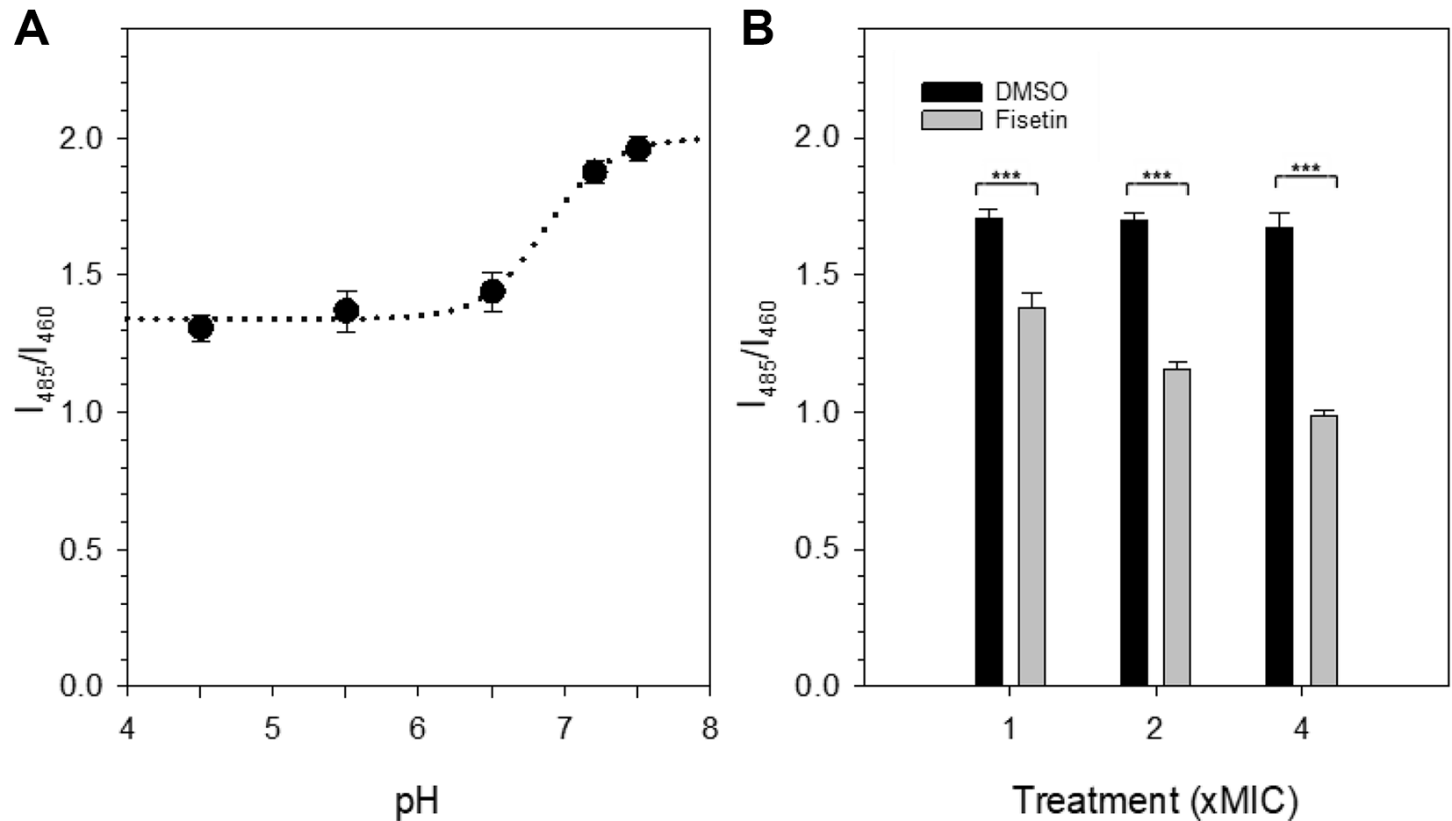
Measurement of ratiometric pH changes of *C. albicans* cells. (**A**) A pH standard curve was established using BCECF, AM-labeled cells, combined with an intracellular pH calibration kit consisting of buffers A (pH 4.5), B (pH 5.5), C (pH 6.5), D (pH 7.5), and PBS (pH 7.2), along with 10 μM valinomycin and 10 μM nigericin. Ratiometric pH measurements employing BCECF (I_485_/I_460_) were conducted. The ratio of I_485_/I_460_ was plotted against pH, and the data presented depict the mean of four measurements ± standard deviation at the 4-h time point. (**B**) BCECF, AM-labeled cells in PBS (pH 7.2) were incubated with either DMSO (black bars) or fisetin (gray bars) at concentrations equivalent to 1x (78 μg/ml), 2x, and 4x the MIC for 1 h at 30°C. Ratiometric pH measurements using BCECF (I_485_/I_460_) were then performed after 8 min, and the presented data are the mean values from quadruplicate measurements, with the standard deviation indicated. ***p < 0.001.

To investigate the impact of fisetin on intracellular pH, a pH standard curve using BCECF, AM was established. Initially, *C. albicans* cells treated with BCECF, AM were incubated in pH calibration buffers ranging from pH 4.5 to 7.5. These buffers included the addition of valinomycin and nigericin. Valinomycin, a potassium-specific ionophore, facilitates the transport of potassium ions across lipid membranes down the electrochemical potential gradient [33], thereby equalizing intracellular potassium concentrations. Meanwhile, nigericin, a proton-specific ionophore, ensures uniform pH across the entire cellular volume. Subsequently, ratiometric pH measurements were performed using BCECF (I_485_/I_460_), and a pH standard graph was plotted, as depicted in Fig. 6A. The standard curve, representing I_485_/I_460_ against pH, exhibited a sigmoidal shape with a rapid increase observed at pH 7.0, corresponding to the pKa of BCECF. Since BCECF predominantly localized within vacuoles in BCECF, AM-treated *C. albicans* cells, achieving intracellular pH equilibrium required time, particularly with BCECF present in both vacuoles and the cytoplasm after incubation of BCECF, AM-labeled cells in the calibration buffer containing valinomycin and nigericin.

During preliminary experiments, an observation was made that the values of I_485_/I_460_ increased with the incubation time of BCECF, AM-treated *C. albicans* cells in the calibration buffer containing valinomycin and nigericin. After 1 h of incubation, I_485_/I_460_ ranged from 0.65 to 1.08 across pH values from 4.5 to 7.5. Fluorescence microscopic analysis confirmed the presence of deep green fluorescent vacuoles and green fluorescent cytoplasm. Subsequently, I_485_/I_460_ measurements were conducted hourly, and the values nearly stabilized after 4 h of incubation, as illustrated in Fig. 6A. The sigmoidal shape remained consistent in the standard curve across all measurements. Following this, BCECF, AM-treated cells were exposed to various concentrations of fisetin, with DMSO serving as the control, and ratiometric pH measurements were conducted (Fig. 6B). The I_485_/I_460_ values of 1x, 2x, and 4x DMSO-treated control cells were 1.71, 1.70, and 1.68, respectively, corresponding to pH values of 6.5, 6.5, and 6.4. These values align with total intracellular pH values contributed by BCECF mostly in vacuoles, considering that BCECF was found only in vacuoles. These results suggest that the pH in the cells remained relatively stable under the influence of DMSO. In contrast, the I_485_/I_460_ value of 1x fisetin-treated *C. albicans* cells was 1.4, indicative of a pH of 5.9. Furthermore, those of 2x and 4x fisetin-treated *C. albicans* cells were 1.2 and 1.0, respectively, corresponding to pH values lower than 4.5, falling outside the reliable range on the standard curve due to the proximity of BCECF's pKa to 7.0. The pH values correspond to the total intracellular pH values contributed by BCECF in cytoplasm and vacuoles. While the exact pH values induced by fisetin treatment were not confirmed, the data unequivocally indicate a significant reduction in intracellular pH, as supported by consistently low I_485_/I_460_ values (*p* < 0.001).

In conclusion, the data depicting lowered intracellular pH (Fig. 6B), along with the presence of several cells uniformly stained green throughout the cytoplasm, with no distinct vacuolar boundary in BCECF, AM-stained cells (Fig. 5F), strongly suggest that fisetin has a significant impact on membrane and vacuolar permeability.

### Fisetin Induces Membrane Hyperpolarization in *Candida* Cells

Membrane potential plays a pivotal role in facilitating the uptake of various molecules against their concentration gradients through symporters and antiporters in diverse cell types. Typically, cells in higher organisms maintain an internal environment characterized by a negative charge relative to their exterior, a phenomenon known as the cell's membrane potential. Depolarization of cells leads to a reduction in their internal charge, making it less negative or more positive. DiBAC_4_(3), identified as a lipophilic anionic dye, readily permeates depolarized cells, binding to lipid-rich intracellular components. Consequently, increased depolarization leads to a greater influx of the anionic dye DiBAC_4_(3), leading to an increase in fluorescence. Conversely, hyperpolarized cells, where the membrane potential becomes more negative than the resting membrane potential, typically exhibit reduced DiBAC_4_(3) uptake. This is because the dye is more likely to exit the cells, causing a decrease in fluorescence.

Given the observed decrease in intracellular pH in fisetin-treated *C. albicans* cells, it was explored whether fisetin treatment induces changes in the membrane potential of these cells. Hence, DMSO- or fisetin-treated *C. albicans* cells were incubated with the lipophilic anionic dye DiBAC_4_(3), and fluorescence intensity was measured using a spectrofluorometer.

**Fig. 7:**
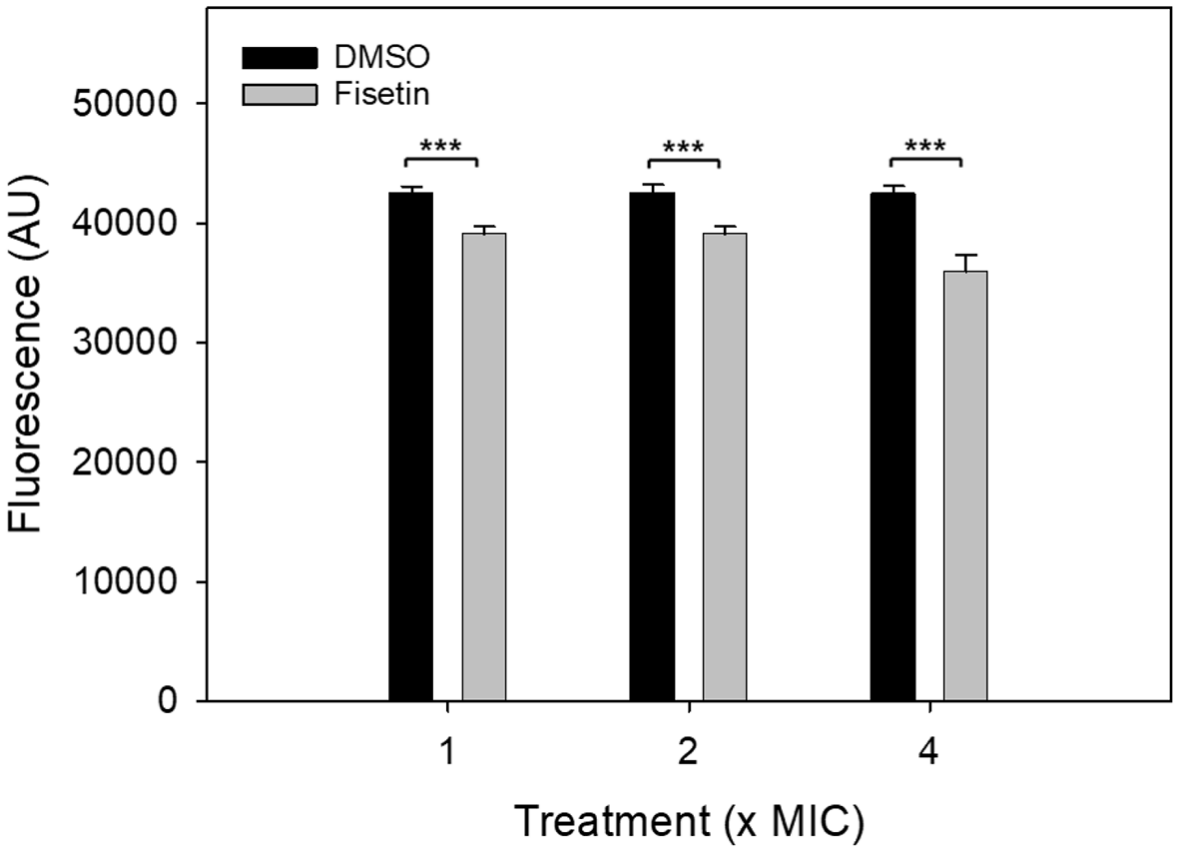
Effect of fisetin on membrane potential. *C. albicans* cells (1 × 10^7^ cells) were incubated with fisetin at concentrations equivalent to 1× MIC (78 μg/ml), 2× MIC, or 4× MIC for 30 min. A DMSO control was included for each treatment. The cells were then incubated with PBS (pH 7.2) containing 20 μg/ml DiBAC_4_(3), and the fluorescence intensity (AU, arbitrary units) was measured using a spectrofluorometer. The data represent the mean of quadruplicate measurements obtained from two independent experiments. Each value is presented as the mean ± standard deviation. Statistical significance is denoted as ***p < 0.001.

The data unequivocally demonstrate that a 30-min fisetin treatment induces a substantial decrease in DiBAC_4_(3) uptake in *C. albicans* cells, clearly indicating membrane hyperpolarization. At both 1x (78 μg/ml) and 2x MIC levels, there was an approximately 8% reduction in DiBAC_4_(3) uptake (Fig. 7). This effect was most pronounced at the 4x MIC level, resulting in a 15% reduction. These findings were all statistically significant (*p* < 0.001) and underscore fisetin's considerable impact on the membrane potential of *C. albicans* cells.

The crucial protein in the yeast cell membrane responsible for maintaining membrane potential is the proton pump Pma1 (P-type H^+^-ATPase), creating an electrochemical gradient across the plasma membrane. This gradient is utilized by other transporters to energize the uptake of ions and nutrients [34]. Plasma membrane H^+^-ATPases, considered conserved proton pumps, actively extrude H^+^ from the cytosol, regulating intracellular pH and generating a transmembrane proton-motive force for coupled transport [35]. Concurrently, yeast possess vacuolar V-type proton pump V-ATPases, structurally conserved and functionally versatile proton pumps found in all eukaryotes. These pumps are located in lysosomes, vacuoles, and endosomes, sustaining an acidic lumenal pH crucial for various cellular processes [36].

## Discussion

The obtained results present compelling evidence of fisetin's antifungal effects, as manifested by significant crystal violet uptake, propidium iodide accumulation, nucleotide and protein leakage, intracellular acidification, and hyperpolarization of *C. albicans* cells. These results collectively suggest that fisetin disrupts the cell membrane and related functions, ultimately contributing to its antifungal efficacy.

Fisetin's actions on cell membrane integrity are postulated as potential mechanisms. Firstly, fisetin may perturb the lipid composition and organization of the cell membrane, potentially compromising its functions. The observed binding of fisetin to ergosterol (Table 2) suggests a potential impact on its antifungal effectiveness against *C. albicans*. Analogous to amphotericin B, fisetin binds to ergosterol, forming pores or channels in the fungal cell membrane. This action elevates membrane permeability, enabling ions and other molecules to leak out of the cell, thereby disrupting the electrochemical balance crucial for the cell's survival.

Secondly, the observed alterations in intracellular pH are likely closely linked to changes in membrane potential. If fisetin inhibits the plasma membrane proton pump Pma1, responsible for pumping protons (H^+^) out of the cell, it could lead to an accumulation of protons (H^+^) inside the cell. This accumulation is likely to result in a decrease in cytoplasmic pH, leading to acidification.

Consequently, this could hinder the movement of other ions, such as potassium ions (K^+^), through the potassium-selective outward ion channel TOK1, ultimately causing hyperpolarization. Such hyperpolarization can have far-reaching effects on various cellular functions, potentially impacting nutrient uptake and pH regulation.

Thirdly, fisetin’s antifungal action may be enhanced by its robust interaction with a specific component of the *C. albicans* cell surface. Autofluorescence on the exterior of *C. albicans* cells (Fig. 4F) indicates a specific interaction between fisetin and the cell surface, suggesting fisetin's affinity for certain molecules. Similarly, the observation of fisetin coating *C. albicans* cells, resulting in aggregates (Fig. S1), further supports a robust interaction with specific components of the cell surface. Reports on amphotericin B's interaction with the cell membrane also align with these observations: Amphotericin B adsorbs to the cell membrane, orienting parallel to the lipid surface, destabilizing the membrane by sequestering ergosterol to the bilayer surface [37]. Moreover, amphotericin B is reported to exist as a large aggregate in the proximity of fungal membranes, extracting ergosterol from them [38]. Likewise, fisetin is reported to bind to human serum albumin through electrostatic interactions with an initial hydrophobic association [8]. All this information supports a strong interaction of fisetin with specific components of the cell surface, likely altering the physical properties and behavior of the cells.

In conclusion, this study not only enhances the understanding of fisetin's mechanisms but also lays the foundation for further investigations into its potential applications in treating fungal infections. The multifaceted impact of fisetin on the cell membrane, intracellular pH, and cell surface interactions highlights its promising role as an antifungal agent, opening avenues for future research and clinical exploration.

## Supplemental Materials

Supplementary data for this paper are available on-line only at http://jmb.or.kr.



## Figures and Tables

**Table 1:** Minimum Inhibitory Concentrations (MICs) of fisetin against *Candida* species.

	MIC (μg/ml)	
	Fisetin	Amphotericin B
*C. albicans* SC5314 (ATCC MYA-2876)	78	1
*C. glabrata* ATCC 2001 (KCCM 50044)	39	1
*C. krusei* ATCC 6258 (KCCM 11426)	39	0.5
*C. parapsilosis* ATCC 22019	19.5	1

The MICs of fisetin against various *Candida* species were determined using the CLSI M27-A3 method.

**Table 2:** Ergosterol binding assay.

	MIC (μg/ml)			
	Fisetin		Amphotericin B	
	24 h	72 h	24 h	72 h
RPMI	78	156	1	1
RPMI + ergosterol	78	312	1	4

The susceptibility of *C. albicans* SC5314 to fisetin was tested using a modified CLSI M27-A3 method in the absence or presence of 400 μg/ml ergosterol. MIC values were determined after 24 h and 72 h of incubation, respectively.
