# Supplementary material for: Fisetin-Mediated Perturbations of Membrane Permeability and Intracellular pH in Candida albicans
Source: J Microbiol Biotechnol. 2024 Jan 10;34(4):783–94. doi: 10.4014/jmb.2311.11027 (PMC11091701; doi:10.4014/jmb.2311.11027)
Supplement: Supplementary file 1 [file jmb-34-4-783-supple.pdf]

J. Microbiol. Biotechnol.
https://doi.org/10.4014/jmb.2311.11027

Supplementary Figure

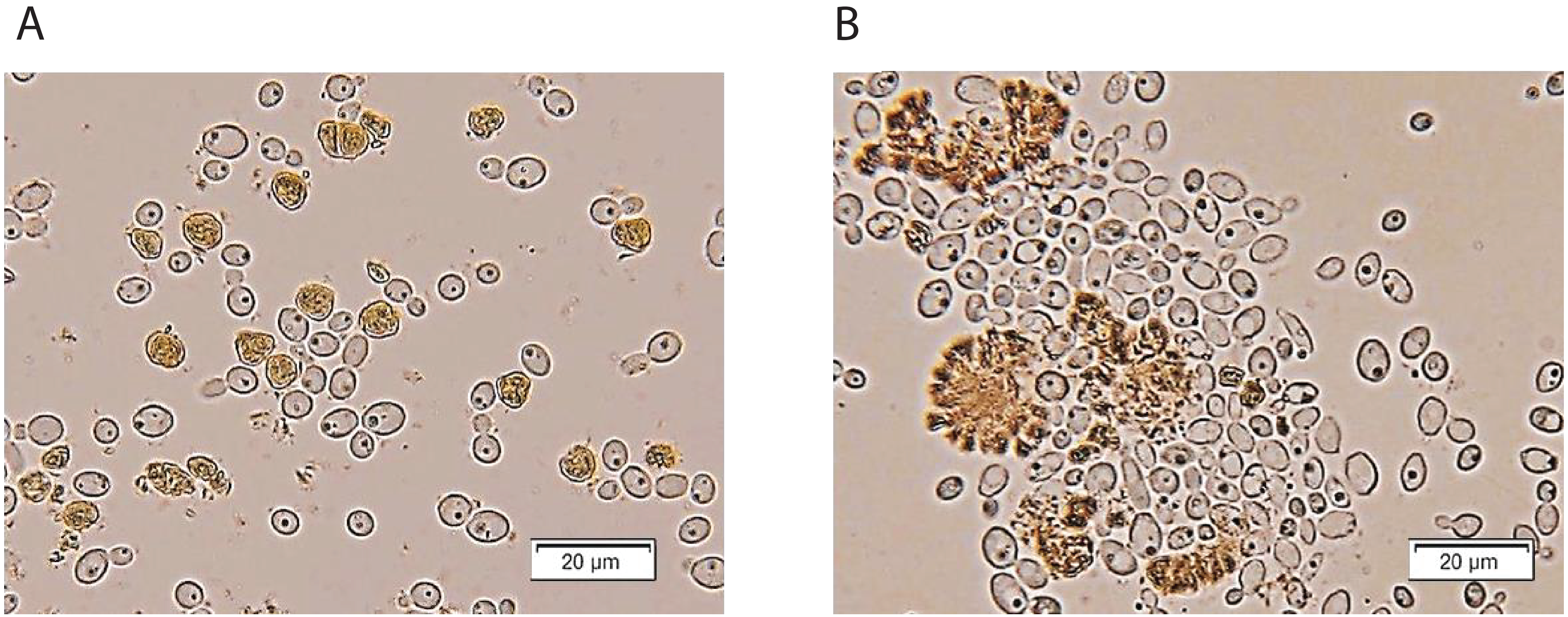

Fig. S1. Interaction of fisetin with *C. albicans* cell surface components.

C. albicans cells were treated with fisetin at its 1x MIC **(A)** and 2x MIC **(B)**, respectively, for 1 hour, revealing the interaction of fisetin with cell surface components. The resulting fisetin-coated cells **(A)** and aggregates **(B)** were examined under a light microscope. Scale bar: 20 μM.
